# Refractory Lupus Nephritis in a Woman With Systemic Lupus Erythematosus on Immunosuppressive Therapy: A Case Report

**DOI:** 10.7759/cureus.96309

**Published:** 2025-11-07

**Authors:** Christopher H Goss, Ahmad Al Tumizi, Atul Singh

**Affiliations:** 1 Internal Medicine, Charleston Area Medical Center, Charleston, USA

**Keywords:** chronic kidney disease (ckd), general internal medicine, general nephrology, lupus nephritis, systematic lupus erythematosus

## Abstract

Systemic lupus erythematosus (SLE) is a multisystem rheumatologic condition that can affect nearly every organ. Recognition of lupus nephritis, a subset of SLE, is critical to preserve organ function. In this case report, we highlight a case of combined class III and V lupus nephritis in a young woman with SLE who subsequently progressed to chronic kidney disease (CKD) stage IIIb while on treatment with immunosuppressants, despite adequate medication compliance. A 24-year-old African American woman with a history of SLE presented to our hospital with severe nephrotic-range proteinuria and new-onset lower extremity edema. She had refractory lupus, as evidenced by persistent proteinuria despite six months of immunosuppressive therapy. She was treated initially with steroids and azathioprine; however, over time her lupus flared and her kidney function worsened despite treatment with mycophenolate, steroids, and eventually voclosporin. To date, she has had several lupus flares while on treatment and appears to have progressed toward CKD stage IIIb.

## Introduction

Systemic lupus erythematosus (SLE) is a rheumatologic condition with a spectrum of multisystem organ disease. The prevalence of lupus in the general population appears to be approximately 20 to 150 cases per 100,000 [1]. Lupus nephritis represents a subtype of glomerulonephritis and is one of the most severe organ manifestations of lupus, involving inflammation of the kidneys at the glomerular level. There are reported cases of systemic lupus presenting with lupus nephritis as the initial manifestation of the disease; however, the more common presentation typically includes fatigue, fever, myalgias, weight loss, arthritis, arthralgias, mucocutaneous manifestations such as a facial malar rash, and various forms of vasculitis [2]. It is also worth noting that renal involvement occurs in approximately 50% of lupus patients [2]. SLE is known as the "great imitator" because it can present in multiple ways and mimic other diseases, both rheumatologic and non-rheumatologic. In addition to lupus, nephrotic syndromes encompass a variety of clinical and laboratory findings resulting from damage to the glomerular filtration barrier, leading to protein loss in the urine. A urine protein excretion rate of 3.5 grams per 24 hours is characteristic of nephrotic syndromes in general [3]. Membranous nephropathy is a subtype of nephrotic syndrome characterized by a diffusely thickened glomerular basement membrane (BM) on light microscopy and subepithelial deposits on electron microscopy [3]. Deposition of immune complexes, including IgG and C3, gives a characteristic "spike and dome" appearance on microscopy, leading to localized inflammation that disrupts the integrity of podocytes and the glomerular filtration barrier, resulting in excess protein loss in the urine [4]. Refractory lupus nephritis is defined as the failure to achieve clinical remission of lupus nephritis despite immunosuppressive therapy [4]. It is associated with an increased risk of progression to chronic kidney disease (CKD) and end-stage renal disease [5]. Lupus nephritis occurs in approximately 20-30% of lupus patients, with African American women at the highest risk for treatment-refractory lupus nephritis [6-9].

## Case presentation

This case involves a 24-year-old African American womanwith a past medical history of SLE, diagnosed six years prior to presentation, and recurrent urinary tract infections, who presented to her rheumatologist’s office with worsening fatigue and peripheral edema for several weeks. Months prior to this presentation, the patient was CKD stage I with a baseline creatinine of 0.8-1.3 mg/dL, a GFR greater than 60 mL/min/1.73 m², and a urine protein-to-creatinine ratio of 1.625 g/g. She was sent to the emergency department (ED) by her rheumatologist for further evaluation.

On presentation to the ED, she was found to be newly hypertensive at 164/104, with additional vitals including a heart rate of 78 beats per minute, a respiratory rate of 16 breaths per minute, a temperature of 37.5 °C, and an oxygen saturation of 99% on room air. Physical examination was significant for +3 bilateral lower extremity edema. She was previously diagnosed with SLE at 18 when she had polyarthritis, myalgias, and a skin rash. Throughout the six years since diagnosis, she had a few episodes of lupus flare-ups, for which she had been treated with tapering doses of steroids. The patient did not tolerate hydroxychloroquine due to its side effects of skin rash and urticaria. She was subsequently started on azathioprine, which led to a transient improvement in proteinuria from 3.5 g/g to 1.6 g/g and stabilization of creatinine over a 12-month period, suggesting partial remission. However, over the following years, she experienced recurrent flares characterized by rising proteinuria and intermittent AKI episodes.

Review of her medical record revealed a history of recurrent urinary tract infections, the most recent episode occurring two months prior to admission, complicated by AKI with a peak creatinine of 2.1 mg/dL. Her creatinine on this admission was 2.2 mg/dL, with a blood urea nitrogen (BUN) of 27 mg/dL, indicating progression to CKD stage IIIb. Urinalysis was positive for proteinuria, hematuria, and leukocyturia, with no evidence of urinary tract infection. Two weeks prior to admission, her urine protein-to-creatinine ratio was approximately 8413 mg/g, which had increased to greater than 15 g/g at presentation. Serologic testing was positive for anti-dsDNA, ANA, and anti-Smith antibodies. Her complement levels showed C3 of 99 mg/dL and C4 of 10 mg/dL. The patient’s nephrotic-range proteinuria was suspected to result from an underlying glomerulopathy or podocytopathy. A summary of laboratory findings at the time of admission is provided in Table 1.

**Table 1:** Key laboratory and serologic findings at the time of admission BUN: blood urea nitrogen, GFR: glomerular filtration rate, C3: complement component 3, C4: complement component 4, ANA: antinuclear antibody, Anti-dsDNA: anti–double-stranded DNA.

Parameter	Result	Reference Range	Units
Blood pressure	164/104	<120/80	mmHg
Heart rate	78	60-100	beats/min
Temperature	37.5	36.1-37.2	°C
Creatinine	2.2	0.6-1.2	mg/dL
BUN	27	7-20	mg/dL
GFR	>60	-	mL/min/1.73 m²
Urine protein/creatinine ratio	>15,000	<200	mg/g
C3	99	90-180	mg/dL
C4	10	10-40	mg/dL
ANA	Positive	Negative	-
Anti-dsDNA	Positive	Negative	-
Anti-Smith	Positive	Negative	-
Albumin	3.4	3.5-5.0	g/dL
Phosphorus	5.1	2.5-4.5	mg/dL
Calcium	8.7	8.5-10.5	mg/dL
Urinalysis	Protein +, Blood +, Leukocytes +	-	-

To rule out renal vein thrombosis, a bilateral renal vein Doppler study was performed, which revealed no acute findings and excluded thrombosis. After a discussion of the risks and benefits with the patient, a kidney biopsy was performed and revealed membranous nephropathy due to a combination of class III and class V lupus nephritis. For her hypertension, losartan, which was started during the inpatient stay, was increased to 100 mg daily to decrease proteinuria. Cardizem was also added, and Lasix was initiated to manage hypervolemia. Pulsed intravenous steroids were administered. For outpatient management, she was prescribed voclosporin, long-term prednisone, and mycophenolate mofetil.

Despite treatment with these medications, the patient appears to have progressed to chronic kidney disease, as evidenced by a baseline creatinine of 1.51 mg/dL averaged over a 10-month period, with a peak creatinine of 2.5 mg/dL during that time, and reported as well as documented compliance with her medications.

## Discussion

This case demonstrates how complex lupus nephritis can be. Even with targeted therapy, this patient’s kidney function declined to CKD stage IIIb. This kind of progression despite treatment shows how some lupus cases remain active despite what would otherwise be considered adequate therapy.

Refractory lupus nephritis indicates that the disease remains active despite patients receiving proper immunosuppressive therapy [4,5]. In this case, antibodies, including anti-dsDNA, antinuclear antibody (ANA), and anti-Smith, were persistently positive, indicating that the autoimmune process remained active and was affecting the patient’s kidneys. These antibodies form immune complexes that damage the glomeruli and activate complement, even when other lupus symptoms improve [4].

This patient had class III/V focal proliferative and membranous lupus nephritis. This combination often leads to heavy protein loss and worse kidney outcomes than either class alone, as noted in this patient [6].

According to the Kidney Disease: Improving Global Outcomes (KDIGO) 2019 guidelines, treatment for lupus nephritis usually starts with mycophenolate or cyclophosphamide added to corticosteroids [5]. Despite proper immunosuppressive medications, about one-third of patients fail to reach remission. The AURORA-1 trial showed that adding voclosporin, a newer calcineurin inhibitor, can improve kidney response and lower the degree of proteinuria [10]. Even newer drugs do not always stop disease progression; genetic factors, complement activity, and podocyte injury may all play a role in why some patients remain refractory [10].

This patient’s intolerance to hydroxychloroquine was a major contributing factor to her presentation. Hydroxychloroquine helps control lupus activity and reduce flares, so intolerance likely made disease control more difficult. Despite steroids, mycophenolate, and voclosporin, her proteinuria worsened, showing that inflammation and microvascular injury may still have been active.

These challenges highlight the need for care plans built around each patient’s specific situation. Treatment choices should consider antibody patterns, medication tolerance, comorbidities, and prior therapies. Ongoing collaboration between rheumatology and nephrology is key, especially for adjusting therapy, managing side effects, and determining when to escalate treatment.

Future research should focus on identifying early warning signs for refractory lupus nephritis and mapping the immune pathways that keep the disease active. Targeting those pathways may help clinicians act sooner and prevent irreversible kidney damage [10]. Although medication adherence was documented, recurrent urinary tract infections may have precipitated flares and transient renal injury, leading to worsening CKD progression. CYP3A5 polymorphisms impacting calcineurin inhibitor metabolism may have modified her therapeutic response to calcineurin inhibitors [11]. Hydroxychloroquine intolerance may also have contributed to poor disease control in this patient since this medication is associated with lower flare rates [12].

Biologic therapies, including rituximab (anti-CD20) and belimumab (anti-BLyS monoclonal antibody), hold promise for refractory lupus nephritis by reducing disease activity and proteinuria in cases of conventional agent failure [13,14]. Additional studies should be conducted to clarify their efficacy in combined class III/V lesions.

## Conclusions

In this report, we highlighted a case of a young woman with a history of lupus who, despite being on seemingly appropriate medical therapy, presented with worsening renal involvement. This case illustrates the complexity of rheumatologic conditions such as lupus and the critical importance of diagnosing both the disease and any renal involvement that may subsequently develop.

A comprehensive evaluation, including serologies and kidney biopsy, was performed to determine why her disease was refractory. She had persistent proteinuria despite adherence to guideline-directed medications and was found to have refractory class III/V lupus nephritis. She also had a difficult course with recurrent urinary tract infections and intolerance to hydroxychloroquine, which likely increased her risk for treatment failure. More research is needed to fully understand the complex interplay between rheumatologic conditions and the kidneys, to improve therapeutic approaches, and to develop targeted medications that enhance outcomes for patients living with SLE. Future studies should also focus on identifying predictors of resistance to therapy and the impact of genetic and pharmacologic variability on immunosuppressive response in patients refractory to conventional regimens.
